# Functional tic-like behaviours during the COVID-19 pandemic: Follow-up over 12 months

**DOI:** 10.3389/fped.2022.1003825

**Published:** 2023-01-09

**Authors:** Adriana Prato, Federica Saia, Maria Chiara Milana, Miriam Scerbo, Rita Barone, Renata Rizzo

**Affiliations:** ^1^ Child and Adolescent Neurology and Psychiatric Section, Department of Clinical and Experimental Medicine, Catania University, Catania, Italy; ^2^ Department of Cognitive Sciences, Psychology, Education and Cultural Studies, University of Messina, Messina, Italy

**Keywords:** tourette syndrome, functional tics, COVID-19, social media, functional movement disorders

## Abstract

**Background:**

Functional tics are included in the wide spectrum of functional movement disorders (FMDs). Their distinction from organic tics is challenging because they both phenomenologically present common features. During the COVID-19 pandemic, there has been an increase in functional tic-like behaviours in vulnerable children and adolescents after social media exposure. This study explores the phenomenology and course of a cohort of newly diagnosed functional tic-like behaviors.

**Methods:**

We analysed clinical data of 243 patients affected by tic disorders collected at outpatient Tourette Clinic, Child and Adolescent Neurology and Psychiatry Unit, Catania University. Among the clinical cohort with functional tic-like behaviors, we evaluated the clinical course of symptoms at follow-up visits after 6 and 12 months.

**Results:**

Among the cohort of 243 patients referred for evaluation at our centre, 11 were diagnosed with functional tic–like behaviours. The majority of participants with functional tic–like behaviours were female with a mean age of 15 years old and presented an explosive symptom's onset. At follow-up visit after 12 months, patients with functional tic-like behaviors showed a significant variation in the severity of tics and anxiety symptoms. Conversely, depressive, and obsessive-compulsive symptoms did not significantly differ during the follow-up.

**Conclusion:**

Our data suggest that several characteristics in clinical course and their phenomenology can help clinicians to distinguish functional tic–like behaviours from organic tics. Our results also suggest a better outcome for tics and anxiety symptoms respect on other comorbidities. A prompt diagnosis and management not only of tics but also comorbidities are recommended, as generally conventional pharmacotherapy for tics does not have positive effects on these patients.

## Introduction

Functional movement disorders (FMDs) are conditions in which affected patients develop abnormal movements that are incongruous with known “organic” diseases, often associated with psychological stressors or social influences (1). FMDs appears to be a common presentation within the spectrum of functional neurological disorders (FNDs). It has been widely described in the literature how FMDs can be triggered by psychosocial stressors and adverse experiences (2). For too long, functional diagnoses were seen as diagnoses of exclusion, a last resort when no “medical” explanation could be found (3). Functional tics appears to be a rare presentation within the spectrum of FMDs. Furthermore, functional tic-like behaviors (FTLBs) can be hard to distinguish from organic tics typical of Tourette Syndrome (TS), making this differential diagnosis challenging, especially for nonexpert clinicians (4). In addition, it could be difficult to distinguish functional tics from organic tics because they share also phenomenological traits and may coexist in the same patient (5). During the global pandemic caused by COVID-19, the social contexts for children and young people have been significantly dissimilar to what they had experienced before. In this period, characterized by an implementation of lifestyle disruptions and social distancing, the digital technology use of various social media platforms such as Tik-Tok and Instagram quickly expanded. In the context of reporting an increase of new cases of FMD during the global pandemic, the advancement of tic-like behaviors while watching movements with analogous features on social media was noted and termed with the alliterative term, “TikTok tics” (6). It was hypothesized that this unusual presentation is related to lockdown, the psychological pressure of the COVID-19 pandemic and social media exposure in vulnerable children and adolescents (7). Many of the “influencers functional tic sufferers describe following on social modelling” media with tic-like behaviours, suggesting “disease is occurring due to social media contagion (7)”. Furthermore, the term mass social media-induced illness has also been introduced for this new type of mass sociogenic illness spread solely *via* social media (8). The phenomenology of FTLBs on social media platforms (e.g., TikTok, Instagram, YouTube) was recently assessed in two studies, demonstrating a high level of coprophenomena, female predominance, context-dependence, aggression toward others, and self-injurious behaviors (9, 10).

Many reports regarding FMDs have been conducted on pediatric samples, but data are still restricted. Considering the general abrupt growth in tics during the recent pandemic period, in this study we aim to explore the phenomenology and course of newly diagnosed FTLBs in our outpatient Tourette Clinic, from June 2021 to June 2022. The study also aimed to describe the clinical differences between patients with functional tics and patients affected by TS or CTD. Lastly, we evaluated in patients affected by FTLBs the clinical course of symptoms at follow-up visits after 6 and 12 months.

## Methods

### Study design

This study was conducted at the Child and Adolescent Neurology and Psychiatry Unit of the Department of Clinical and Experimental Medicine of Catania University. A total of 243 patients referred to and followed by our outpatient Tourette Clinic for tics were enrolled in the Childhood Tic Disorders Clinical Database. Eligible participants were patients aged 6–18 years of age with a sudden onset or increase of possible tics or FTLBs, recruited from June 2021 to June 2022 at the outpatient Tourette clinic of the Child and Adolescent Neuropsychiatry Unit at Catania University Hospital. Prior to enrolment, all parents provided written informed consent, and the subjects assented when possible. Demographics and clinical data from all participants were reviewed. Data collection was made in the context of a standardized research assessment and included demographic variables such as age and sex, a detailed information about the course and phenomenology of patients’ tics, presence of comorbidities, possible treatment approaches, social media exposure, possible triggers, and precipitating events. Before inclusion in the study, all patients were screened with the Schedule for affective disorders and Schizophrenia for School age children—present and lifetime (Kiddie-SADS-PL) to rule out primary psychiatric disorders considered as criteria of exclusion. Then, all patients underwent neuropsychiatric evaluation for TS and related comorbidities. The Kiddie-SADS-PL is a semi-structured interview tool developed by Kauffman et al. (11) that can be used in children and adolescents aged between 6 and 18 years (11). Considering the clinical evaluation at baseline from a team of pediatric neuropsychiatrists with solid expertise in tic disorders and FNDs, participants were classified as affected by TS/CTD or FTLBs, according to DSM-V criteria. Among the clinical cohort, modifications in symptoms severity were further evaluated after 6 and 12 months.

### Clinical assessment

All participants underwent the first assessment at baseline (T0). The Wechsler Intelligence Scale for Children (WISC-IV) was administered to quantify the intelligent quotient (IQ) of children (12). Patients were also assessed according to Yale Global Tic Severity Rating Scale (YGTSS), Children's Yale-Brown Obsessive-Compulsive Scale for Children (CY-BOCS), Multidimensional Anxiety Scale for Children (MASC), Child Depression Inventory (CDI). The YGTSS is a clinician-rated scale used to evaluate the motor and phonic tic severity through the examination of number, frequency, duration, intensity, and complexity of tics (13). The CY-BOCS is a semi-structured interview rating the severity of obsessions and compulsions arising over the past week across five areas (time, interference, distressing nature, effort to resist, control over obsessions and compulsions) (14). All patients compiled the MASC, a scale that assessing anxiety symptoms (15) and the CDI, a self-report instrument that evaluated possible depressive symptoms in pediatric patients (16). Finally, patients with FTLBs were examined for tics and potential associated comorbid disorders after 6 months (T1) and after 12 months (T2). Modifications in symptoms severity were evaluated by the difference in the administered scales. Those patients who manifested a decrease no less of 25% in rating scales have been considered as “responders”.

### Statistical analysis

Data were analyzed using SPSS software (SPSS, Inc., Chicago, IL, USA, IBM, Somers, NY, USA). Clinical variables of patients are summarized using means and standard deviations (SD) for continuous data or count (%) for categorical data. We assessed the distribution of quantitative variables to determine their deviation from the normal distribution (Shapiro–Wilk test). Since the distribution of the rating scales was not normal, we assessed the time-points by non-parametric methods. Specifically, we computed the variation between the values at the two time points (first consultation and follow-up visit at 12 months). Clinical outcomes among T0 and T2 were also evaluated to discriminate responders’ patients, who showed a reduction at least 25% in scores (17). A *p*-value < 0.05 was considered statistically significant.

## Results

### Sample description

Within 12 months (June 2021 to June 2022), a total of 243 patients aged 7–18 years (mean age = 12.3 ± 3.5) were enrolled in the Childhood Tic Disorders Clinical Database. Functional tics were recognized in eleven children, and primary tic disorders diagnosed in the other 232 children, according to the fifth edition of the Diagnostic and Statistical Manual of Mental Disorders (DSM-5) (18). Participants with FTLBs were more likely female (female = 72.7%) and presented a mean age of 14.8 ± 2,6 years old. Four (36.4%) patients had family or childhood history of TS or another tic disorder. The onset of FTLBs was rapid and sudden in eight (72.7%) patients during the pandemic, with a mean age at onset of 14 ± 2.6 years. Of the eleven participants with FTLBs, five (45.4%) presented a history of mild simple motor or vocal tics in childhood. Functional tics were correlated with premonitory urges (PUs) prior to tics by only three patients (27.3%). Seven (63.6%) participants with rapid onset FTLBs had a varied repertoire of complex motor and vocal tics with no rostro caudal progression at onset, including echolalic and coprolalic like-behaviors. Of the eleven patients with FTLBs, four (36.4%) endorsed exposure to a single social media influencer showing tic-like behaviors, while four (36.4%) reported prior personal exposure to individuals affected by motor and/or vocal tics. Clear precipitating factors were identified in five (45.4%) of patients with functional tics, including family- and virtual schooling related emotional distress. A subgroup of patients (63.6%) had other associated functional neurological disorders (non-epileptic seizures, functional motor symptoms) in addition to tics. Demographic and clinical characteristics of our sample with FTLBs are summarized in Table 1. Compared with FTLBs, patients with primary tic disorders (*n* = 232, female = 10.8%) were younger at diagnosis (mean age = 12.2 ± 3.5) and at symptom onset (mean age = 7 ± 2.7). Among the individuals diagnosed with TS/CTD, only a minor percentage reported the presence of complex motor and vocal tics (*n* = 21, 9.0%), echolalia (*n* = 25, 10.8%), coprolalia (*n* = 21, 9%), copropraxia (*n* = 15, 6.5%) and other FNDs (*n* = 15, 6.5%). Furthermore, a prior exposure to influencer on social media with tic-like behaviours was reported only in 21 (9.0%) patients with TS/CTD, while the presence of a clear precipitating factor was documented in 20 (8.6%) of them. Regarding treatment approaches in TS/CTD, 34 patients had received CBT (14.6%) and 68 patients take anti-tic medications (29.3%).

**Table 1:** Clinical features of patients with functional tic-like behaviours (FTLBs).

Variable	Case 1	Case 2	Case 3	Case 4	Case 5	Case 6	Case 7	Case 8	Case 9	Case 10	Case 11
Gender	F	F	F	M	F	M	M	F	F	F	F
Age, years	18	17	17	13	13	13	13	11	13	17	18
Age at onset	18	17	17	12	13	12	12	11	11	15	16
Family history of Tic disorders	Yes	No	Yes	No	No	No	Yes	No	Yes	No	No
Abrupt functional tics onset	Yes	Yes	Yes	Yes	Yes	Yes	Yes	Yes	No	No	No
Presence of complex harm/hand motor tics	Yes	Yes	Yes	Yes	Yes	Yes	Yes	Yes	Yes	Yes	Yes
Presence of complex vocal tics	Yes	Yes	Yes	Yes	Yes	Yes	Yes	No	No	No	No
Presence of complex motor and vocal tics	Yes	Yes	Yes	Yes	Yes	Yes	Yes	No	No	No	No
Premonitory sensations	No	No	No	No	No	Yes	Yes	No	No	Yes	No
Presence of precipitating event/trigger	Yes	Yes	No	Yes	No	Yes	No	No	Yes	No	No
Presence of echolalia	Yes	Yes	Yes	Yes	Yes	Yes	Yes	No	No	No	No
Presence of coprolalia	Yes	Yes	Yes	Yes	Yes	Yes	Yes	No	No	No	No
Presence of copropraxia	Yes	Yes	Yes	Yes	Yes	Yes	Yes	No	No	No	No
Other functional neurological disorders	Yes	No	No	Yes	No	Yes	Yes	Yes	Yes	Yes	No
Pharmacological treatment	Yes	Yes	Yes	Yes	No	Yes	Yes	No	Yes	No	Yes
Cognitive behavioural therapy (CBT)	Yes	Yes	Yes	Yes	No	Yes	No	No	Yes	No	Yes
Prior exposure to tics	Yes	No	Yes	No	Yes	No	No	No	Yes	No	No
Prior exposure to social media influencer with tic-like behaviors	Yes	Yes	Yes	No	Yes	No	No	No	No	No	No

During the follow up period, all participants with FTLBs have been treated with CBT, and two of them also started a pharmacological treatment with benefits. These two patients reported a slight reduction in anxiety symptoms, a better quality of sleep, and a partial relief of functional tics.

### Neuropsychiatric evaluation

Participants presented a mean IQ of 85.5 (±18.1). In general, the functional tic group compared to CTD/TS presented at baseline higher scores on the YGTSS (mean total score 32.5 vs. 18.0), CYBOCS (mean total score 16.7 vs. 7.4), CDI (mean total score 10.7 vs. 9.8) and MASC (mean total score 52.1 vs. 17.1). Among the patients with FTLBs, while there was observed a decrease in rating scales (YGTSS, MASC, CDI, CYBOCS) from initial consultation to 12-month follow-up, the detected change was not statistically significant. Mean YGTSS score at first consultation was 32.5 (SD 14.9) and improved at 6-follow-up visit (mean = 29.7; SD 9.9) and at 12-month- follow-up (mean = 24.4; SD 11.7), with a mean total decrease of 8.1 points (25.0%) (*p* = 0.1813) (Table 2). Furthermore, 45.5% (*n* = 5) of patients achieved at least 25% reduction in YGTSS scores from baseline (T0) at T2. Conversely, patients with FTLBs didn't show a significant variation in the severity of obsessive-compulsive symptoms, as evaluated by CYBOCS between the first visit (mean score at T0 = 16.7, SD 8.3) and the follow-up visit after 6 months (mean score at T1 = 16.7; SD 7.4) and 12 months (mean score at T2 = 16.8; SD 15.7) (*p* = 0.9867) (Table 2). CDI scores were in normal range at baseline and during the follow-up visits (*p* = 0.7179) (Table 2). However, there was a improvement from T0 to T2 in anxiety symptoms on the MASC, with a mean reduction of 16.5 points (31.8%) on the MASC total score (mean score at T0 = 52.1, SD 23.3; mean score at T1 = 43.3; SD 19,2; mean score at T2 = 35.6; SD 20.1) (0.09141) (Table 2).

**Table 2:** Yale global Tic severity rating scale (YGTSS), children's Yale-brown obsessive-compulsive scale for children (CY-BOCS), multidimensional anxiety scale for children (MASC), child depression inventory (CDI) outcome in patients affected by FTLBs.

	First consultation	6-month-follow-up	12-month-follow-up	Mean change from baseline after 12 months	Confidence interval, 95%	*p*-value
YGTSS total score	32.5 (SD 14.9)	29.7 (SD 9.9)	24.4 (SD 11.7)	8.1 (SD 3.2)	[−4.0031, 19.8213]	0.1813
CYBOCS total score	16.7 (SD 8.3)	16.7 (SD 7.4)	16,8 (SD 15,7)	0.1 (SD 7.4)	[−11.285, 11.1032]	0.9867
MASC total score	52.1 (SD 23.3)	43.3 (SD 19.2)	35.6 (SD 20.1)	16.5 (SD 3.2)	[−2.9016, 35.8107]	0.09141
CDI total score	10.7 (SD 7.4)	10.9 (SD 6.6)	9.6 (SD 6.5)	1.1 (SD 0.9)	[−5.1185, 7.3003]	0.7179

## Discussion

This study investigates the clinical features of pediatric patients affected by FTLBs. So far, a very limited number of studies have evaluated the phenomenology of functional tics before the pandemic caused by COVID-19 (5, 19–21). Functional tics are rarely reported in patients affected by tic disorders, compared with other functional symptoms (22, 23).

During the COVID-19 pandemic, a dramatic growth in rapid onset FTLBs has been observed (4, 6, 24–27). The global pandemic caused by COVID-19 has been an important source of stress and onset of neuropsychiatric disorders for people throughout-the-world, causing an increased request for mental health services (24). During the pandemic, it was also observed a worsening of chronic neurological and psychiatric diseases even in those without COVID-19 infections (9). Both pandemic-related restrictions on social gatherings and increase in use of social media platforms have been implicated as precipitating factors in the increase of FTLBs (4). Furthermore, there has been a dramatic growth in tic-related videos on social networks (TikTok, Instagram, Youtube). In this context characterized by a a rise of newly diagnosed FTLBs, Hull et al. (1) suggested that functional tics developing after watching analogous movements on social networks be named as TikTok tics. Recently, a few studies have been conducted on patients affected by functional tics, even after social media exposure. In a study conducted on patients presenting with FTLBs (*n* = 20) compared with patients affected by TS or other primary tic disorder, Pringsheim et al. (24) founded several distinguishing clinical features for the diagnosis of FTLBs, including the rapid onset of symptoms, female gender, complex vocalizations and coprophenomena, social media exposure. Their participants with FTLBs had higher YGTSS total tic and impairment scores, and significantly higher total symptoms scores on the MASC and CDI (24). In another prospective cohort study, clinical features of TS patients (*n* = 24) were compared to those of participants with FTLBs (*n* = 9), despite a small sample size of only thirty-three participants (4). All participants with FTLBs of this cohort reported an abrupt symptom onset, the presence of premonitory sensations prior to tics, and exposure to social media with #Tics and #Tourettes (4). Instead, Paulus et al. (25) compared clinical variables between 13 patients with FTLBs and 13 patients with TS and founding several clinical characteristics allowing to distinguish between the two group, some of which discriminated completely (ie, abrupt symptom onset, lack of symptom fluctuations, symptom worsening in social contexts) and some nearly perfectly (ie, predominantly complex movements involving trunk/extremities) (25). Furthermore, Hull and Parnes (6) described six girls, with an explosive onset of FTLBs after exposure to social media influencers. Within this cohort, sudden presentation without a previous history of similar movements, presence of “tic attacks”, other associated FMD, uncommon triggers, lack of PU, and incapacity to suppress movements were supportive of the diagnosis of FTLBs (6). In another retrospective review of 34 children presenting with sudden onset tic-like movements, the authors also showed in this case-series a higher prevalence of pali/echo/copro-phenomena and psychiatric and neurodevelopmental comorbidities, particularly anxiety and autism spectrum disorders (ASD) (26). Moreover, Han et al. (27) reported an important increase in the percentage of functional tics during the COVID-19 pandemic (10.6% in 2020, 36% in 2021), highlighting differences in clinical features between patients with functional tics and patients with primary tic disorders to aid diagnosis. Conversely, a subgroup of these patients (18.2%) reported exposure to social media content involving tics prior to presentation of FTLBs (27). Previous studies regarding patients affected by FTLBs are summarized in Table 3. Instead, recent original research described the course and treatment of rapid onset FTLBs in adolescents (*n* = 20) and adults (*n* = 9) previously reported in two case series (4, 24) and showed a better prognosis in adolescents respect on adult patients with FTLBs (28). The authors reported at 6-month follow-up visit in the adolescent group a mean total decrease of YGTSS total tic score of 15.3, and described that the most used treatment approaches were selective serotonin reuptake inhibitors (SSRIs) and CBT (28). Finally, a recent multicentre international study confirmed substantial clinical differences between primary tic disorders and FTLBs (29).

**Table 3:** Summary of studies on functional tic like-behaviours before and after COVID-19 pandemic.

Authors	Patients (FTLBs/PTD)	Female	Age at onset	Family history of PTD	Prior PTD	Abrupt onset	PUs	Precipitating trigger	Echolalia	Coprolalia	Copropraxia	Other functional neurological disorders	Prior exposure to social media
**Before COVID-19 pandemic**													
Mejia (2005)	17/155	34.8%	N.A.	38.7%	100%	0%	N.A.	64.7%	N.A.	N.A.	N.A.	N.A.	∕
Baizabal-Carvallo (2014)	9/273	55.6%	34.1	0%	0%	N.A.	22.2%	N.A.	N.A.	N.A.	N.A.	100%	∕
Demartini (2014)	11/0	27.3%	37.2	0%	0%	100%	18.2%	90.9%	0%	0%	0%	72.7%	∕
Ganos (2016)	13/0	30.8%	25.3	0%	100%	76.9%	69.2%	76.9%	38.5%	53.8%	7.7%	38.5%	∕
**After COVID-19 pandemic**													
Pringsheim and Martino (2021)	9/24	100%	15.3	N.A.	22.2%	100%	100%	N.A.	N.A.	67%	N.A.	N.A.	100%
Pringsheim et al. (2021)	20/270	95%	13.9	N.A.	15%	100%	N.A.	N.A.	N.A.	N.A.	N.A.	N.A.	100%
Paulus (2021)	13/13	38.5%	15.31	7.7%	0%	92.3%	76.9%	N.A.	38.5%	38.5%	53.8%	N.A.	100%
Hull (2021)	6/0	100%	14.2	0%	0%	100%	33.3%	83.3%	N.A.	33.3%	N.A.	66.7%	100%
Han (2022)	22/163	100%	13.8	14%	27%	100%	N.A.	81.8%	N.A.	77%	45%	N.A.	18.2%
Buts (2022)	34/0	94%	13.7	N.A.	44%	100%	62%	N.A.	62%	62%	62%	N.A.	62%

**Figure 1:**
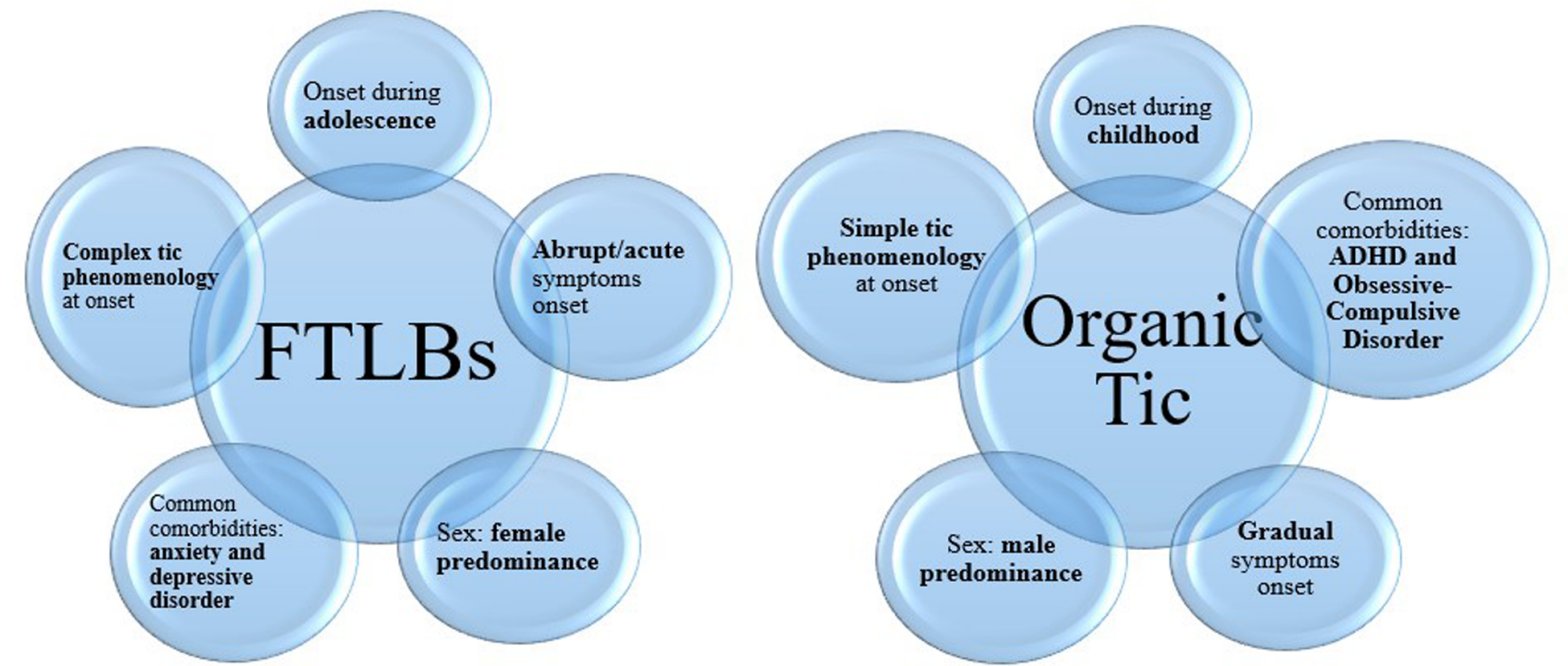
Phenomenological differences between organic tics and FTLBs.

The clinical features observed in our pediatric cohort are like the distinctive characteristics of FTLBs based on previous literature studies, with some exceptions. Common clinical features of our participants with FTLBs include female preponderance, and later age of onset with abrupt and rapid progression of symptoms, such literature studies have just reported (1, 4, 24, 26, 27). A positive family history of tics is reported in 36.4% patients, and 45.4% of them presented a history of mild simple motor or vocal tics in childhood, suggesting the possible overlapping phenomenon between organic tics and FTLBs. The presence of associated FNDs was documented in a subgroup of patients with FTLBs (63.6%), in line with other literature reports (1, 5, 20, 21). Furthermore, in our small cohort affected by FTLBs there is a high percentage of pali/echo/copro-phenomena, compared with other studies (1, 25). Conversely, premonitory sensations were reported by only three patients (27.3%), in contrast to other clinical studies that described a higher percentage of associated PUs (1, 25, 26). Instead, 36.4% of our patients with FTLBs endorsed exposure to a single social media influencer with tic-like behaviors, less respect other recently reported samples (1, 4, 24–26). However, a major increase in the use of social media was reported in our patients with FTLBs; therefore, it is possible to hypothesize that the social media's effects were not fully reported by all our patients. Furthermore, the association with a clear precipitating trigger was outlined in 45.4% of patients with functional tics. Probably, a more structured investigation into this phenomenon could have revealed a higher percentage of precipitating factors. The dramatic course of these abrupt onset FTLBs is reflected by higher symptom severity ratings on the YGTSS, and on other neuropsychological findings evaluating possible comorbidities. In Figure 1, we summarize the main characteristics of both organic tics and FTLBs in our sample.

A significantly greater decrease in tics as measured by YGTSS at 12-month follow-up visit was found in our cohort. Furthermore, participants with FTLBs demonstrated a mean reduction in YGTSS total score of 8.1 (25%). In our cohort, at the follow-up visit we also showed a more significant improvement in anxiety symptoms, with a mean decrease of 16.5 points on the MASC total score. Considering the impact of pandemic Covid-19 on children mental health (30), our findings suggest that both the end of lockdown restrictions and a prompt treatment approach seems effective with management of functional tics and comorbidities. To the best of our knowledge, there is only one recent report describing a follow over 6-months of patients with rapid onset functional tics assessed by YGTSS (28). No other studies have evaluated the course and prognosis of paediatric patients with functional tics, focusing on comorbidities eventually associated. Our research had several limitations. First, the sample size of reported cohort is very small. Second, our study was conducted in a tertiary-care centre, where the majority of TS patients have higher comorbidity and tic severity and, therefore, the results may not apply to patients with a mild form of TS. Conversely, this study had also several strengths, including the long follow-up period, and the evaluation of not only tics but also neuropsychiatric comorbidities. Despite these limitations, our study suggests a better outcome for anxiety symptoms respect on tics and other comorbidities.

## Conclusion

Functional tics has increased during the COVID-19 pandemic in vulnerable children and adolescents. Clinicians should be particularly vigilant in considering the possibility that FTLBs can co-occur in patients with tic disorders who are refractory to multiple therapeutic interventions or who present with dramatic onset of symptoms. Furthermore, functional tics had atypical onset symptoms which might lead the clinicians to make the wrong diagnosis in the early stage of disease. A prompt diagnosis and management not only of tics but also comorbidities are recommended, as these patients are generally not responsive to conventional anti-tic medications, while they may benefit from cognitive behavioural therapies. Despite our results, further trials with more substantial cohorts are necessary to investigate the course and prognosis of patients with FTLBs also affected by other comorbidities.

## Data Availability

The raw data supporting the conclusions of this article will be made available by the authors, without undue reservation.
